# A synthetic three-color scaffold for monitoring genetic regulation and noise

**DOI:** 10.1186/1754-1611-4-10

**Published:** 2010-07-21

**Authors:** Robert Sidney Cox, Mary J Dunlop, Michael B Elowitz

**Affiliations:** 1 Division of Biology, California Institute of Technology, Pasadena, CA, USA; 2 Department of Engineering and Applied Science, California Institute of Technology, Pasadena, CA, USA; 3 RIKEN Systems and Structural Biology Center, Yokohama, Japan; 4 Joint BioEnergy Institute, Lawrence Berkeley National Laboratory, Berkeley, CA, USA; 5 Howard Hughes Medical Institute, California Institute of Technology 1200 E. California Blvd. M/C 114-96 Pasadena, CA 91125, USA

## Abstract

**Background:**

Current methods for analyzing the dynamics of natural regulatory networks, and quantifying synthetic circuit function, are limited by the lack of well-characterized genetic measurement tools. Fluorescent reporters have been used to measure dynamic gene expression, but recent attempts to monitor multiple genes simultaneously in single cells have not focused on independent, isolated measurements. Multiple reporters can be used to observe interactions between natural genes, or to facilitate the 'debugging' of biologically engineered genetic networks. Using three distinguishable reporter genes in a single cell can reveal information not obtainable from only one or two reporters. One application of multiple reporters is the use of genetic noise to reveal regulatory connections between genes. Experiments in both natural and synthetic systems would benefit from a well-characterized platform for expressing multiple reporter genes and synthetic network components.

**Results:**

We describe such a plasmid-based platform for the design and optimization of synthetic gene networks, and for analysis of endogenous gene networks. This network scaffold consists of three distinguishable fluorescent reporter genes controlled by inducible promoters, with conveniently placed restriction sites to make modifications straightforward. We quantitatively characterize the scaffold in *Escherichia coli *with single-cell fluorescence imaging and time-lapse microscopy. The three spectrally distinct reporters allow independent monitoring of genetic regulation and analysis of genetic noise. As a novel application of this tool we show that the presence of genetic noise can reveal transcriptional co-regulation due to a hidden factor, and can distinguish constitutive from regulated gene expression.

**Conclusion:**

We have constructed a general chassis where three promoters from natural genes or components of synthetic networks can be easily inserted and independently monitored on a single construct using optimized fluorescent protein reporters. We have quantitatively characterized the baseline behavior of the chassis so that it can be used to measure dynamic gene regulation and noise. Overall, the system will be useful both for analyzing natural genetic networks and assembling synthetic ones.

## Background

### Regulatory networks

Synthetic biology requires the assembly of regulatory networks encoded in DNA [1-4]. Such networks are designed from qualitative or empirically fitted models of the individual genetic components [5-11], because detailed quantitative measurements [12,13] of these components and their *in vivo *interactions are often lacking. In many cases, the behavior of designed genetic networks differs significantly from initial model predictions.

It would be helpful to have a tool for characterizing the quantitative behavior of both natural and synthetic genetic networks. Here we present a three-color genetic reporter that can be used to monitor dynamic gene expression in single bacterial cells. This "three-color scaffold" is contained on a single DNA construct. The system has been designed to minimize spurious interactions between reporters, maximize signal, and support modular additions. We show that interactions between the reporters are minimal when controlled by different transcription factors. This tool can measure multiple network properties in parallel to characterize multi-component systems.

### Design and characterization

We followed four principles in the sequence design of the three-color scaffold. (1) *Biocompatibility*. The scaffold must be genetically stable and non-toxic to the cells carrying it. (2) *Distinctness*. It must be possible to completely separate the fluorescent signals of the three genetic reporters. (3) *Independence*. The genetic expression of each reporter must be made as independent as possible without spurious cross talk between components. (4) *Modularity*. To make the three-color scaffold generally useful for natural and synthetic biological applications, it must be straightforward to change the various genetic elements.

The construct contains a set of three operons (transcriptional units), each containing a single reporter gene (see Methods and Additional File 1 for more details). Strategically placed unique restriction sites allow modification and expansion of these operons. For the protein coding sequences of each operon we chose three fast maturing, monomeric, and spectrally separable fluorescent proteins: Cerulean CFP [14], Venus YFP [15], and Cherry RFP [16]. The promoter sequences contain the polymerase and transcription factor binding sites [17]; unique restriction sites flank each promoter. To ensure that the operons are genetically independent, multiple transcriptional terminators [18,19] are placed between each operon. The operons are arranged in alternating orientation, with RFP pointing opposite to YFP (the two genes converging) and YFP pointing opposite to CFP (the two genes diverging). Transcriptional read-through is therefore possible only if the RFP promoter reads through three terminators, the oppositely oriented YFP gene, and into the CFP gene.

**Figure 1:**
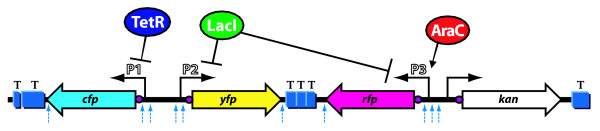
**The scaffold design**. The three reporters are controlled by promoters responsive to: tetracycline/aTc (*cfp*), lactose/IPTG (*yfp*), and both lactose/IPTG and arabinose/L-ara (*rfp*). Terminators are blue boxes denoted "T," RBSs are small purple circles, unique restriction sites are blue dashed-arrows, and promoters are small black arrows. Each fluorescent operon is shown as a colored block arrow, while the kanamycin antibiotic resistance is shown as a white block arrow. The three transcription factors TetR, LacI, and AraC are shown regulating the three fluorescent genes in a simple genetic network: TetR represses *cfp*, LacI represses both *rfp *and *yfp*, and AraC activates *rfp*. The scaffold sequence is in Additional File 1.

We demonstrate the response of the scaffold using three synthetic promoters regulated by three inducible transcription factors (Figure 1). TetR repressor regulates the *cfp *gene at the P1 promoter, the LacI repressor regulates both the *rfp *and *yfp *genes at the P3 and P2 promoters (respectively), and the activator AraC additionally regulates the *rfp *gene at the P3 promoter. Combinatorial promoters that accept multiple genetic inputs similar to P3 are ubiquitous in genomes and are useful for creating synthetic networks [20-22]. We use chemical inducers corresponding to these three transcription factors to vary the activity of each promoter and quantitatively characterize the basic response of the three-color scaffold to each input.

### Noise

Measurements using fluorescent reporter proteins in individual microbes [6,8,9,20,23-30] have helped to quantify the interaction between genetic noise [24,31] and network structure. To demonstrate its utility as a genetic tool, we employ the three-color scaffold to analyze noisy fluctuations of a transcription factor that regulates two target genes simultaneously.

There are many ways to detect regulatory interactions, but context-preserving tools for distinguishing the directionality and causality of regulatory connections within a larger network are lacking. In the engineering field of system identification theory, white noise is often used as an input to characterize system response [32]. Analogously, noise in gene expression can reveal regulation [23,33]. Noisy expression of a regulatory protein will filter through to downstream genes, causing correlations in gene expression levels. The cross correlation function, which measures time-dependent correlations, can be used to quantify the time it takes noise to propagate through a regulatory connection.

Previously, we showed that the maximum cross correlation between a repressor and its target gene occurs with a delay due the time required for a transcriptional event to result in a functional repressor [23]. Here we consider a more general case, where only a subset of the network elements can be measured, and show that noise can still be used to infer network structure. By measuring two downstream targets of an unobserved regulatory factor we show that co-regulation can be inferred without directly measuring (or perturbing) the state of the unobserved factor. This method can be directly applied to regulation systems where either the regulatory factor itself is unknown, or where directly perturbing the regulation is impossible (no known inducer or mutants) or undesirable (pleiotropy).

## Results

### Scaffold design and construction

We designed and built the three-color fluorescent reporter scaffold (Figure 1, sequence in Additional File 1) using custom DNA synthesis to fulfill the design principles (see Methods for additional descriptions of the fluorescent protein properties and microscopy). (1) Biocompatibility: The scaffold was genetically stable and non-toxic to cells carrying it. We minimized the DNA length (4 kb), used a plasmid with a stable low copy origin of replication (SC101), and silently mutated rare codons and restriction sites from the protein coding sequences. We placed the reporter genes under the control of three tightly regulated promoters [34]. (2) Distinctness: fluorescent reporter proteins have recently been engineered for high signal and low crosstalk. We chose three fast maturing, monomeric proteins: Cerulean CFP, Venus YFP, and Cherry RFP. These three reporters are known to have well separated excitation (433, 515, and 587 nm respectively) and emission (475, 528, and 610 nm respectively) peaks, which allow clean separation of their signals with an appropriate choice of fluorescent filters. We minimized the microscope spectral crosstalk to be less than 0.1% and correctable to within 0.01%. (3) Independence: We wished to detect both strong and weak genetic signals simultaneously, with the ability to watch them change over time in single cells. We used multiple transcriptional terminator sequences (Methods) to isolate the reporter genes. (4) Modularity: Because the scaffold is one small continuous piece of DNA, it can be easily moved between different vectors. Unique restriction sites are upstream of each promoter, at the end of the promoter, and at the end of each protein-coding region. This allows for replacement of promoters, insertion of additional genes into each operon, and modification of the reporter protein coding sequences. Small genetic elements (e.g. translation signals and protein tags) can easily be changed by adding them as extra sequences on the end of PCR primers for each region. Unique restriction sites separate the three operons and can be used to add more operons or construct complex networks.

### Three-color induction

We used the three reporters to monitor regulation by the transcription factors AraC, LacI, and TetR. Each transcription factor can be controlled independently by the addition of chemical inducers L-ara, IPTG, and aTc, respectively. Repressors TetR and LacI control separate promoters (P1 and P2) expressing CFP and YFP, respectively (Figure 1). The combinatorial LacI/AraC regulated P3 promoter [34] expresses RFP. We describe below how changing the inducer conditions characterizes the response of the three-color scaffold to these three transcription factors.

**Figure 2:**
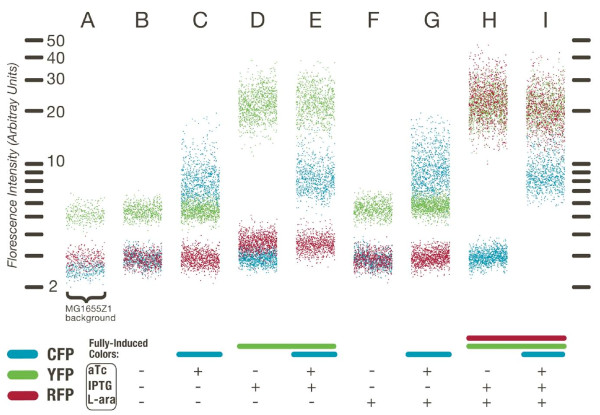
**Operons provide independent control of gene expression**. The framework is measured on a low-copy plasmid in wild type *E. coli *strain MG1655Z1 containing endogenous AraC levels, and high levels of the repressors LacI and TetR. Fluorescence microscopy snapshots were taken of 500-1000 cells under each combination of saturating inducer concentrations. Each cell within a population is represented by three dots--one for each color--in order to show the cell-cell variability in each condition. For clarity, each cell within a measurement condition is shown using a single dot for each color; individual points are offset horizontally to enable visualization of the entire data set. (A) The autoflourescence of *E. coli *MG1655Z1 without any fluorescent proteins. (B, C, D, E, F, G, H and I) The response of each reporter to different combinations of the three inducers; each column is one condition with the expected induced colors shown as a bar below.

Two inducible genes are independent when induction of one gene does not affect the expression of the other. We first verified that each of the three inducers (L-ara, IPTG, and aTc) only affected the expression of genes regulated by their corresponding transcription factor (not shown). We then characterized the scaffold in single *E. coli *cells containing chromosomal copies of each transcription factor (strain MG1655Z1 described in [21]) using quantitative fluorescence microscopy (Figure 2). In this strain, all three fluorescent proteins are repressed prior to addition of inducers. Compared to the mean cellular autofluorescence (measured in cells without the plasmid, Figure 2A) cells carrying the plasmid showed very weak (~5%), but detectable, leaky expression of the *yfp *and *cfp *genes (Figure 2B). The autofluoresence of *rfp *was so low, and the P3 promoter so tightly regulated, that we could not detect any difference in red fluorescence when the scaffold was introduced into a strain without induction. We tested whether the transcriptional units in the scaffold plasmid could be induced independently by using combinations of saturating inducer concentrations. We found the mean expression of *cfp *and *yfp *to be independent (Figure 2C, D, and 2E), as well as the mean expression of *cfp *and *rfp *(Figure 2C, F, and 2H), indicating that there was no significant transcriptional read-through from *rfp *into the downstream co-oriented gene *cfp*. The expression of both *yfp *and *rfp *increased in the presence of IPTG (Figure 2D and 2E). Only *rfp *was induced by L-ara (Figure 2F, J, H, and 2I)--but only when IPTG was also present. We performed an additional control experiment where *rfp *was placed under the control of the cI repressor from λ phage to verify that it could be controlled independently from *yfp *(not shown). These results show that the design of the scaffold provides genetic isolation, enabling independent control of the three reporter genes.

The combinatorial LacI/AraC-regulated promoter controls *rfp *as an asymmetric AND [21] gate (Figure 2B, D, F, and 2H), with the repressor LacI acting as the dominant transcription factor. Expression was undetectable when induced with L-ara alone (Figure 2B, C, F and 2G). In the absence of L-ara, IPTG induced expression only slightly (25%) above the autofluorescence level (Figure 2D and 2E). In the presence of L-ara, IPTG induced expression to 40× the autofluorescence level (Figure 2H and 2I). These results show that LacI and AraC control expression at promoter P3 combinatorially, with three distinct output expression levels.

### Gene expression noise and static correlation

Fluctuations in plasmid copy number, cell-wide transcriptional/translational activity, or growth rate should change expression of all genes in a correlated fashion. Deviations from this basal correlation can suggest additional regulation. We first verified that only the appropriate inducers affected the total genetic noise in each color (Additional File 2). Under conditions in which all fluorescent proteins were induced (Figure 2I), we calculated correlation coefficients for each pair of fluorescent reporters (Table 1). Both the linear (Pearson) parametric and rank (Spearman) non-parametric correlation coefficients [35] gave similar results in each case. We also calculated the partial correlation coefficients (Methods), which indicate the degree of extra correlation between two variables when the third is held fixed. Only *yfp *and *rfp *exhibited significant partial correlation, and correlation between *yfp *and *rfp *was consistently higher than the correlation between either *yfp *and *cfp *or *rfp *and *cfp*. The additional correlation between *yfp *and *rfp*, as compared to *cfp*, was consistent with noisy LacI co-regulation.

**Table 1:** Multi-color noise correlations reveal co-regulation.

	Correlation	Partial Correlation	Rank Correlation	Partial Rank Correlation
*ρ (cfp, yfp)*	0.43 ± 0.06	0.23 ± 0.10	0.40 ± 0.06	0.19 ± 0.07
*ρ (yfp, rfp)*	0.85 ± 0.02	0.83 ± 0.02	0.85 ± 0.01	0.71 ± 0.03
*ρ (cfp, rfp)*	0.37 ± 0.07	-0.07 ± 0.11	0.36 ± 0.06	-0.04 ± 0.08
*ρ (yfp, rfp) MG1655*	0.94 ± 0.01	0.93 ± 0.01	0.94 ± 0.01	0.93 ± 0.01
*ρ (yfp, rfp) ΔlacI*	0.48 ± 0.13	0.02 ± 0.18	0.51 ± 0.14	0.16 ± 0.18
*ρ (yfp, rfp) ΔlacO*	0.38 ± 0.12	-0.14 ± 0.09	0.19 ± 0.07	-0.14 ± 0.07

**Figure 3:**
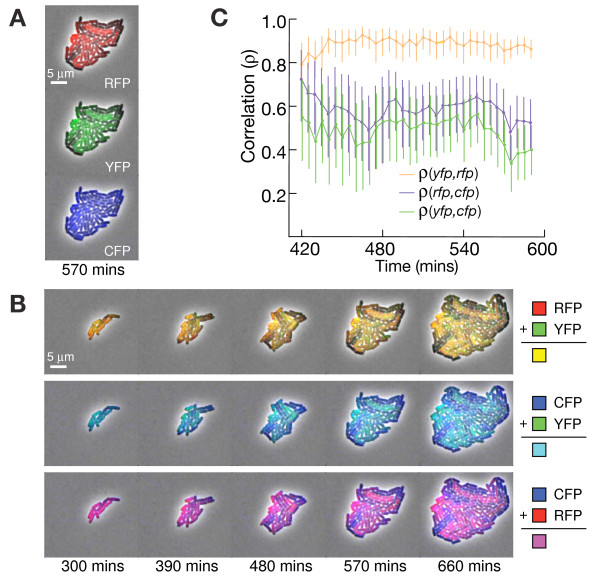
**Time-lapse images**. We monitored levels of *cfp*, *yfp*, and *rfp *expression from the construct shown in Figure 1 during growth in a microcolony of *E. coli *MG1655. This strain has low levels of LacI and no TetR, leaving *cfp *constitutively active. We grew the microcolonies on agarose pads, using arabinose as a carbon source to ensure AraC induction. (A) The pseudo-colors indicate the expression levels of *rfp *(red), *yfp *(green), and *cfp *(blue). (B) Two-color images, made by adding pairs of images colored as in A, show how each pair of colors varies over time in single cells. Cells appearing yellow, the combination of red and green, reveal the correlation between *yfp *and *rfp *due to LacI co-regulation. (C) Correlation between pairs of colors as a function of time calculated across all cells in the microcolony, the error bars represent 90% confidence intervals (Methods). These average correlations persist over several hours of microcolony growth.

We hypothesized that noise in LacI might be used to detect the co-regulation of its targets. We performed three additional experiments to confirm that the added correlation was indeed due to LacI repression at the *yfp *and *rfp *promoters (Table 1). First, we measured the same system in wild-type *E. coli *MG1655, which contains approximately 300× lower endogenous levels of the repressor LacI (10 protein copies/cell in MG1655 [36] as opposed to 3 × 10^3 ^protein copies/cell present in the Z1 strain [34]) and no TetR repressor. In this strain, induction of LacI was not necessary to observe *yfp *and *rfp *expression (Figure 3A). As expected, repression was weaker with the lower concentration of LacI repressor. The correlation between *yfp *and *rfp *was larger than with high LacI levels (ρ = 0.94 versus ρ = 0.85), due to the increased noise in LacI concentration at low protein copy number. Second, we measured the correlation between *yfp *and *rfp *in a strain containing a deletion of the entire *lac *operon (*ΔlacI*). Third, we switched the LacI/AraC controlled *rfp *promoter with a constitutive promoter containing no LacI operators (*ΔlacO*). In both the second and third experiments LacI regulatory connections were disconnected, and the extra correlation between *yfp *and *rfp *disappeared (Table 1); this confirmed that the increased correlation between *yfp *and *rfp *was due to transcriptional co-regulation by LacI. These results imply that noise in gene expression may reveal transcriptional co-regulation directly in a wild type background strain (MG1655) without requiring any external perturbation or induction.

### Time-lapse noise correlation

The extra correlation observed between *yfp *and *rfp *due to LacI co-regulation should persist over time in growing cells. We measured three-color fluctuations in a growing microcolony with time-lapse microscopy (Figure 3, Additional Files 3 and 4). We used the scaffold in the wild type MG1655 strain (no TetR, low LacI), and grew microcolonies with arabinose (L-ara) as the carbon source to ensure AraC induction. When averaged over the cells present in the microcolony, the time-series revealed strong correlation (Figure 3C) between *yfp *and *rfp*. Thus, noisy regulation by LacI correlates the fluctuations of its target genes *yfp *and *rfp *during several generations of microcolony growth.

Fluctuations in LacI should simultaneously affect its two target promoters. The cross correlation function measures correlations one signal and a second signal shifted in time relative to the first [23]. When two signals are highly correlated, but there is a delay between the first and second signal, the cross correlation function can be used to identify the lag (delay time) that maximizes the correlation. In particular, if noise in a protein takes some time to propagate through a regulatory network, the signal will be correlated with the original noise source offset by this lag. The cross correlation function reaches a maximum at the time-delay for which the correlation between the two signals is highest (Methods).

**Figure 4:**
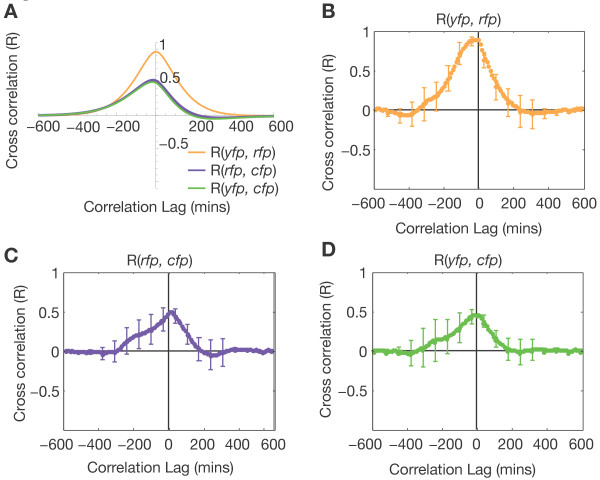
**The cross correlation function reveals regulatory connections**. (A) A stochastic mathematical model of noisy LacI co-regulation (Methods) predicts a large, symmetric *yfp-rfp *cross correlation function and smaller asymmetric *rfp-cfp *and *yfp-cfp *cross correlations. (B) Experimental cross correlation between *yfp *and *rfp *averaged over 5 microcolony movies. The error bars show the standard error between movies. (C) Experimental cross correlation between *rfp *and *cfp*. (D) Experimental cross correlation between *yfp *and *cfp*.

If two genes are regulated by a noisy factor simultaneously, their cross correlation function should be symmetric with maximum at zero. In contrast, if the factor regulates one of its targets indirectly (e.g., by repressing an activator of one target) the cross correlation function would be asymmetric due to the delay of indirect regulation [23,33]. A stochastic mathematical model (Methods) of the regulatory network shown in Figure 1 (in the absence of the TetR repressor) exhibits symmetry in the cross correlation between *yfp *and *rfp *(Figure 4A). The experimental cross correlation between *yfp *and *rfp *(Figure 4B) agrees with the qualitative features of this model; the peak correlation between *yfp *and *rfp *occurs at zero lag, indicating that the co-regulation is mediated instantaneously by LacI. The time-lapse experiment verifies that LacI simultaneously correlates the expression of its two target genes.

The cross correlations between the LacI regulated (*yfp *and *rfp*) genes and the constitutive control (*cfp*) gene are similar to each other (Figure 4C and 4D): For each the maximum cross correlation occurs at zero lag, the maximum cross correlation (R = 0.45) is much smaller than the maximum cross correlation between *yfp *and *rfp *(R = 0.95), and the cross correlation has an asymmetric shape. This asymmetry occurs due to fluctuations in LacI. Too see this, consider the extrinsic fluctuations in the expression of all genes. If a cell has a momentary global increase in gene expression at time *t *= 0, the three reporters will be transiently expressed highly--but so will LacI. Once this transient pulse has matured into a functional LacI protein (*t = t_1_*), it will repress its targets and the *yfp *and *rfp *(but not *cfp*) levels will eventually drop (*t = t_2_*). As a result, the correlation between the future (*t = t_2_*) *yfp *and *rfp *levels and the present (*t = 0*) *cfp *level will be less than if *yfp *or *rfp *were unregulated. In other words, the positive half of the cross correlations R(*rfp, cfp*) and R(*yfp, cfp*) will reach zero faster than the negative half. The mathematical model reproduces this asymmetry (Figure 4A) for both R(*rfp, cfp*) and R(*yfp, cfp*). These results show that fluctuations in LacI simultaneously affect only the two target genes (*yfp *and *rfp*) but not the constitutive gene (*cfp*). Thus the three-color reporter can be used to distinguish genes regulated by noisy transcription factors from constitutive ones.

## Discussion

We have developed a genetic construct for measuring dynamic gene expression in single cells using three fluorescent proteins on the same plasmid. We chose three fluorescent proteins that have well characterized spectra and fast maturation times. We strictly minimized any overlap in the excitation and emission spectra of the fluorophores and observed the three genes expressed independently.

Advances in fluorescent protein and imaging technology will permit similar analysis of more than three simultaneous reporters. Additional fluorescent proteins such ultramarine [37] (excitation peak 355 nm, emission peak 424 nm), or far-red [38] (excitation peak 684 nm, emission peak 708 nm) could be used to expand our system. We explicitly chose three monomeric fast-maturing fluorescent proteins with minimally overlapping spectra in order to avoid any false correlations due to spectral overlap of the proteins. Even a very small spectral cross-talk between two reporters would show up as a very strong correlation signature, and we caution against adding fluorescent reporters for noise analysis without ensuring that the detection system is still entirely spectrally distinct. Overlapping spectra may be de-convoluted [39], but any inaccuracy in the correction technique could lead to strong false positive correlations.

Multiple reporters can be spatially separated: either in different regions of the cell, or within differentiated cell types of a multicellular organism. Recently three different fluorescent reporters were used to strikingly label ten different neuron cell types in transgenic mice [40]. Such applications do not require the stringent spectral separation necessary for the noise correlation analysis presented here.

To study natural genetic networks, one or more scaffold promoters (P1, P2, or P3) can be switched to appropriate natural promoters. The activity of multiple promoters could be quantified at the same time by using the promoters provided here as a baseline control. Promoters could be screened for quantitative changes in activity in various environmental conditions. For example, cells carrying a version of the scaffold that monitors three regulated promoters could be measured by fluorescence activated cytometry in different media conditions to determine a particular promoter activity state (e.g. two promoters off, one promoter on). In this way the reporter scaffold can be used to monitor and compare multiple context-sensitive natural promoter responses within the same regulatory network.

The scaffold will aid synthetic network construction and optimization. Previously constructed synthetic networks can be analyzed in the same way as natural networks, by inserting their regulatory promoters into the framework to control expression of the three fluorescent proteins. Monitoring multiple network promoters in this way can provide insight into internal variables of the system that would otherwise be unknown. Alternatively, synthetic gene networks can be built directly into the scaffold reporter backbone by using the unique restriction sites between each genetic element to create transcriptional fusions of network genes. These network genes can be paired with target promoters of the three operons, adding additional operons to build more complex networks as necessary. To test the modularity of specific parts, a synthetic network of transcription factors and promoters could be characterized first individually (e.g., with the transcription factors expressed from inducible promoters) and then again after they are connected.

The noise correlation method complements modern bioinformatic and experimental high-throughput techniques for analyzing natural transcriptional networks: (1) Comparing noise correlation between pairs of uncharacterized promoters versus their correlations with a constitutive promoter can suggest co-regulation--even when the co-regulating factor is unknown. (2) Putative transcription factor binding sites may be analyzed in our three-color reporter scaffold against a confirmed target promoter of the same transcription factor. For example, more than 200 novel CRP binding sites have been predicted based on species comparison [41]; our novel method can test the functionality of these predicted sites. One color could be used for the constitutive control, one color for a confirmed CRP-regulated gene [42], and the final color could be used to test CRP regulation at a predicted site. (3) Many transcription factors, including CRP, regulate as both activators and repressors [43]. Biochemical techniques may reveal transcription factor binding at a promoter, but often do not resolve whether the interaction is activating or repressing. Correlation between two promoters will increase relative to the control when the sign of co-regulation is the same (both activation or both repression) or decrease when the signs differ (one activation and one repression). If the uncharacterized promoter is compared to a promoter with known function the sign of regulation will be clear. (4) Gene expression profiles from high-throughput microarrays can identify regulatory connections by comparing multiple genes across a variety of environmental and genetic perturbations [44]. Unlike microarray approaches, dynamic cross correlation analysis of time-lapse fluctuations is affected by the direction and activity state of regulatory connections between two transcription factors even in the steady state with no environmental perturbation. (5) Finally, time-lapse correlation analysis may reveal regulation by an uncharacterized transcription factor. Though the presence of correlation does not always imply a common direct regulator, it can suggest additional experiments to test for indirect regulatory connections. Uneven propagation of extrinsic noise between a regulated gene and a constitutive control can result in an asymmetric cross correlation function, as seen in the case of LacI regulation in Figure 4C and 4D.

## Conclusion

We present a three-color scaffold for monitoring gene expression, where the individual genes are independent of each other. With this system it is possible to insert three transcription units directly into a plasmid so that they can be analyzed simultaneously using fluorescent protein reporters. Our scaffold provides a biocompatible, distinct, independent, and modular platform for a wide variety of *in vivo *applications. We show that a noise source such as LacI can be used as an input to a synthetic regulatory network, and the propagation of this noise can be monitored to confirm the intended network structure. Correlation analysis of genetic noise can suggest the presence of regulatory connections even when no suitable condition is known--it doesn't require finding a specific mutation (e.g., *ΔlacI*) or chemical signal (e.g., IPTG) to perturb the regulatory interaction in question.

## Methods

### Synthetic DNA design

The 3,518 base-pair scaffold was constructed by total synthesis by DNA2.0 in Menlo Park, CA. The sequence was cloned into the plasmid vector pDrive, and confirmed by automated sequencing. The annotated sequence of the scaffold in plasmid pZS2-123 is supplied (Additional file 1).

Unique restriction sites allow for insertion and swapping of genetic elements between the promoters, protein coding regions, and terminators of the construct. The three fluorescent protein sequences were codon optimized for expression in bacteria. We created a modified codon usage table by averaging the two codon usage frequency tables of Gram-negative *E. coli *and Gram-positive *Bacillus subtilis *[45]. We further modified the averaged codon usage tables, to remove rare (less than 10% usage) codons--including arginine codons (AGG and AGA) that have been shown to be toxic when expressed highly in *E. coli *[46]. Each protein sequence was then back translated into a DNA sequence by sampling each codon in proportion to the frequency of the modified codon usage table. For the *cfp *coding sequence, we used the amino acid sequence of the Cerulean *cfp *variant [14] for back-translation and optimization. For the *yfp *sequence, we used the amino acid sequence of the Venus *yfp *variant [15], and incorporated the mutations of the Citrine *yfp *variant [47]. For the *rfp *sequence, we used the amino acid sequence of the Cherry *rfp *variant [16]. The coding sequences were silently mutated to remove any of the specified unique restriction sites. Double stop codons (TAATAA) were used at the end of all three protein sequences to ensure efficient termination of translation. We used promoters P_LtetO-1 _(P1) to control *cfp*, P_LlacO-1 _(P2) to control *yfp*, and P_lac/ara-1 _(P3) to control *rfp *[34]. To control translation, we used the moderate strength SD8 RBSs for *cfp *and *yfp *[48], and the stronger RBS from gene 10 of phage T7 for *rfp *[49]. These were chosen because the P_lac/ara-1 _promoter is weaker than the P_LtetO-1 _and P_LlacO-1 _promoters. Terminators RNAI and TSAL [50] terminated the transcriptional unit containing *cfp*. Terminators TR2-17 [19], TL17 [51], BS7 [50], and T7TE+ [52] terminated the transcriptional units containing *yfp *and *rfp*.

### Plasmids and strains

The initial synthetic construct was cloned into the modular pZ* expression vector system [34] using the NotI and NheI restriction sites on each end of the scaffold construct. This plasmid system allows for easy swapping of the origin of replication and antibiotic resistance markers. Data for Figures 2 and 3 is from measurement of plasmid pZS2-123, containing: a kanamycin resistance marker; the SC101 origin of replication, and the promoters described above. To measure the correlation between *rfp *and *yfp *with the LacI regulation of *rfp *removed (Table 1, *ΔlacO*), we placed the *rfp *gene under the control of the (constitutive due to the absence of cI) P(R) promoter from phage λ [53]:

cccgggcatacgttaaatctatcaccgcaagggataaatatctaacaccgtgcgtgttgactattttacctctggcggtgataatggttgcatgcctagg

This promoter sequence was synthesized and cloned in between the restriction sites XmaI and AvrII to create plasmid pZS2-12R.

We used wild-type *E. coli *MG1655 [54] for the time-lapse experiment (Figure 3, Additional Files 3 and 4), and to measure the correlation between *yfp *and *rfp *from plasmid pZS2-123 in the presence of low levels of LacI (Table 1, MG1655). This strain does not contain the TetR repressor, so *cfp *is expressed constitutively. The MG1655Z1 strain was constructed from the wild type MG1655 strain and the TetR and LacI over-expressing DH5aZ1 [34] strain by P1 general transduction [55]. We used MG1655Z1, which over-expresses LacI from the lacI^q ^promoter, for the characterization of the scaffold response to induction (Figure 2). We also measured the correlation between *yfp *and *rfp *in strain MC4100, which does not contain the LacI gene (Table 1, *ΔlacI*).

It is essential for the fluorescent reporters to mature quickly and uniformly for correlation measurements to be accurate. In our movies the time between cell divisions was 87 ± 25 minutes, corresponding to a protein dilution rate of 7.97 × 10^-3 ^min^-1^. Our scaffold uses the Venus YFP protein and Cherry RFP protein, both of which are known to have maturation times of 15 minutes [15,16]. If one fluorescent protein were to mature much slower than the other, it would create an artificial lag in the cross correlation function and shift the curve horizontally.

### Microscopy

Single-cell measurements were acquired on an Olympus IX-81 inverted fluorescence microscope at 100× magnification, with a Hammamatsu Orca ER CCD camera (2 × 2 binning) using custom microscope acquisition software. Phase-contrast images were acquired to measure cell morphology, position, and image quality. Fluorescent excitation was performed with a Lambda LS Xenon lamp (Sutter Instruments, Inc.) with a liquid light guide and fluorescent filter cubes (Chroma, Inc.) for Cyan/*cfp *(Chroma, #31044v2), Yellow/*yfp *(Chroma, #41028), and Crimson/*rfp *(Chroma, #41027). To prevent photobleaching, all images were collected as ordered exposures of (*rfp*, *yfp*, *cfp*), with minimal light exposure. We verified the fluorescent field provided by the Lambda LS light source and liquid light guide with fluorescent slides (Spherotech, Inc.). The field was found to be extremely flat (std/mean ≈ %3) when centered in all three colors.

In order to check and correct for spectral crosstalk between fluorescent proteins, we constructed plasmids containing each individual fluorescent protein. We measured cells expressing only one of the *cfp*, *yfp*, and *rfp *in all three filter cubes (Table 2). Crosstalk was very small in all cases. The highest magnitude was *rfp *fluorescence in the Yellow/*yfp *channel, which amounted to 0.1% of the detection level in the *rfp *channel. The crosstalk of *cfp *into the Crimson/*rfp *cube was undetectable in our system. All reported data are corrected for this crosstalk using the inverse matrix of Table 2 (see below). Errors in crosstalk measurement could conceivably introduce false correlations into Table 1. To control for this possibility, we repeated all data analysis without the crosstalk correction and found no change in any of the qualitative results of Table 1. These results confirm good spectral separation.

**Table 2:** Spectral crosstalk of three fluorescent reporters.

	CFP	YFP	RFP
Cyan (Chroma #31044v2)	1.0E+00	1.5E-04	1.0E-04
Yellow (Chroma #41028)	5.0E-04	1.0E+00	1.1E-03
Crimson (Chroma #41027)	0.0E+00	1.3E-05	1.0E+00

### Induction experiment

All inducers and chemicals were purchased from Sigma. LB growth medium (Lennox) was used for all cell experiments. All enzymes for plasmid construction and modification were obtained from New England Biolabs. MG1655Z1 cells containing the plasmid pZS2-123 were grown to saturation overnight in LB with 50 mg/mL kanamycin at 37°C and diluted 100× into non-fluorescent M9 minimal medium [56] containing 0.2% glycerol, 0.01% Casamino acids, 0.15 mg/ml biotin, 1.5 mM thiamine, and combinations of the three inducers. Inducer concentrations were 500 mM isopropyl β-D-1-thiogalactopyranoside (IPTG), 100 ng/mL anhydrotetracycline (aTc), 0.1% L-+-arabinose (L-ara). Cells were grown for 3 hours at 32°C to an OD600 of 0.2. For cells induced with aTc, an additional 50 ng/mL of aTc was then added to insure complete induction. Cells were allowed to grow to a final OD600 of 0.3, placed on ice, and measured on 1.5% low melting temperature agarose phosphate-buffered saline slabs in the microscope. For each condition, we acquired approximately 20 fields of cells, totaling 500-1000 cells per condition measured.

### Time-lapse experiment

MG1655 cells containing the plasmid pZS2-123 were grown to saturation overnight in LB at 37°C and diluted 1000× into non-fluorescent M9 arabinose minimal media (as above with 0.2% L-ara instead of glycerol). Cells were grown for 3 hours at 32°C, then diluted 100× and transferred to L-ara media pads made with 1.5% low melting point agarose. These pads were placed inside a glass Wilco dish chamber and sealed. Time-lapse images were acquired at 10 minute intervals in a 32°C temperature controlled chamber.

### Image processing

We used custom software and the Matlab (The Mathworks, Inc.) Image Processing Toolbox to segment the phase contrast images and collect corresponding pixels from each of the three fluorescent images. The program identified individual cells on phase contrast images by progressive watershed thresholds. Shapes were filtered based on morphological properties to eliminate non-cell objects, clumps of cells, and misshapen cells. For each cell, a background value of the minimum pixel contained in the bounding box was recorded for each color. Collected fields of cells were examined by eye to check for errors in segmentation and acquisition. For the time-lapse experiment, we identified cell division events and tracked lineages of cells (lines of daughter and parent cells) during microcolony growth [13,27]. We extracted the data from the segmentation program, subtracted the background autofluorescence value for each cell, and normalized each color with respect to the camera's exposure time for that image. We collected autofluorescence measurements as a daily control. These autofluorescent values were normally distributed (not shown). We then corrected for the spectral crosstalk measured above by multiplying the 3-color data from each strain by the inverse of the spectral crosstalk matrix (Table 2).

We tested for sources of systematic or experimental error. Parabolic fluorescent field correction did not change the qualitative relationships or reduce variation. The overall fluorescence variation between fields of cells remained small: Each frame analyzed was within one standard deviation of the mean over all frames of the same color. Normalization to account for morphological factors such as size and shape did not qualitatively change our results or decrease the observed variation. As a final correction, we removed outlying cells and non-cell objects from the processed data that were more than three standard deviations from the median of the 500-1000 cells. Previous noise measurements have used similar corrections [24,29].

### Static correlation

For each processed data set, we calculated the normalized Pearson and Spearman correlation coefficient between each pair of colors (Table 1). Using these three pairwise correlations, we also calculated the three partial correlation coefficients:

To calculate the errors in correlation and partial correlation coefficients, we uniformly re-sampled 1,000 data sets (bootstrap sampling with replacement) of the same size and recomputed the correlation coefficients for each sample. The errors reported in Table 1 and Figure 3C were determined by the 90% confidence intervals of this bootstrap procedure (by taking the 100^th ^and 900^th ^values of the sorted list of resampled correlation coefficients).

For the time-lapse movies, we calculated the Pearson correlation coefficient for each pair of colors over the cells present in the entire microcolony at each time point (Figure 3C). The error bars on each correlation coefficient became smaller as the number of cells in the microcolony increased. We were able to resolve the correlation coefficients after about 7 hours of growth, corresponding to about 30 cells per microcolony.

### Time-lapse cross correlation

We calculated the cross correlation function between each cell lineage for all three pairs of colors for five microcolonies grown in identical conditions. Each microcolony analyzed contained 62-124 cell lineages. The data shown in Figure 4B, C, and 4D is the mean of the five microcolony cross correlation functions with standard error bars.

The cross correlation between two discrete signals *f(t)*and *g*(*t*) is defined as

Here,  and *N *is the number of time points. This function is normalized to .

We adapted this standard formula to accommodate tree-structured (branching) data as in [23]. The following modified formula incorporates this correction:

Here *N_cells _*is the number of cells at the end of the movie and *k_i _*is the branching point between *f_i _*and *f_i+1_*. As in the non-branched case, the function is normalized by

### Mathematical Model

System dynamics were modeled using stochastic differential equations (Figure 4A). We approximate the system with linear dynamics around a nominal equilibrium point since we expect perturbations due to noise to be small:

where L = LacI, A = AraC, C = CFP, Y = YFP, and R = RFP. Cross correlations are calculated as in [23] with model parameters b = 0.0116 1/min, g_LY _= -0.007 1/min, g_LR _= -0.007 1/min, g_AR _= 0.0007 1/min, W_E _= 6.39x10^-3^, W_L _= 100, W_A _= W_C _= W_Y _= W_R _= 1.

## Competing interests

The authors declare that they have no competing interests.

## Authors' contributions

RSC designed the DNA sequences, performed the experiments and drafted the manuscript. MJD carried out the time-lapse data analysis and mathematical modeling, and helped write the manuscript. MBE conceived of the study, and participated in its design and coordination. All authors read and approved the final manuscript.

## Supplementary Material

Additional file 1 **pZS2-123 annotated sequence**. Sequence of the three-color reporter construct in a 6,806 base-pair plasmid containing a Kanamycin resistance gene and an SC101 origin of replication. Sequence +1 begins at the single NotI restriction site at the beginning of the 3,518 base-pair three color reporter scaffold (the three color scaffold sequence ends at the NheI site). All genetic elements described in the Methods section are annotated as sequence features, along with the primers used for sequence verification. Click here for file

Additional file 2 **Figure S1: Total genetic noise is controlled by induction.** The total genetic noise, calculated as the standard error divided by the mean, is plotted for each of the conditions in Figure 2. Cyan corresponds to noise in *cfp*, yellow to noise in *yfp*, and red to noise in *rfp*. In each case, the noise is maximal in the fully induced state. The noise of each color is only affected by the associated inducer(s): aTc for *cfp*, IPTG for *yfp*, and both IPTG and L-ara for *rfp*. Click here for file

Additional file 3 **Timelapse movie of 3 color network, yellow and cyan channels**. Click here for file

Additional file 4 **Timelapse movie of 3 color network, yellow and red channels**. Click here for file
