# Supplementary figures and images for: A synthetic three-color scaffold for monitoring genetic regulation and noise
Source: J Biol Eng. 2010 Jul 21;4:10. doi: 10.1186/1754-1611-4-10 (PMC2918530; doi:10.1186/1754-1611-4-10)

**Figure S1.**

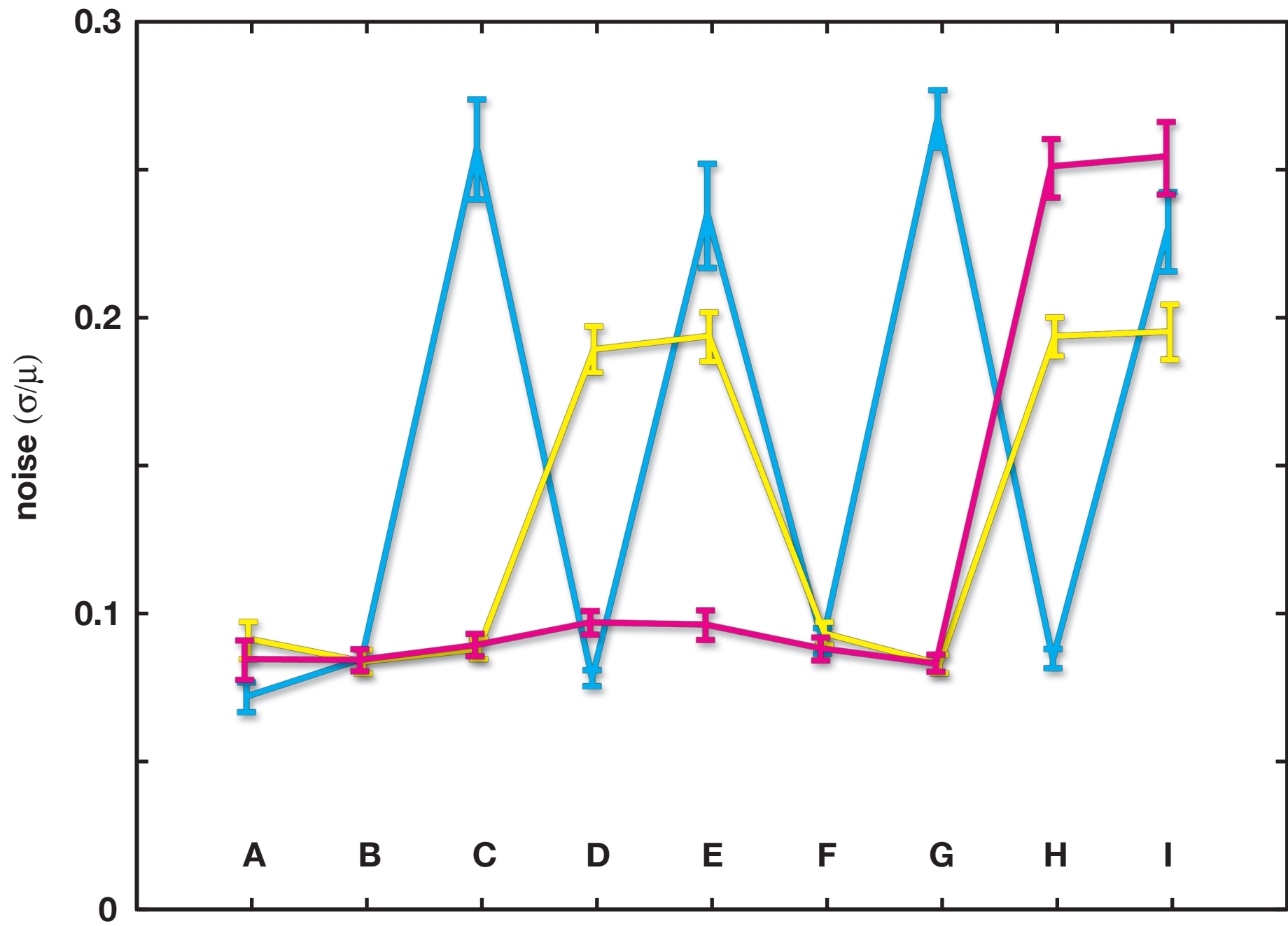

Supplement: Additional file 2 — Figure S1: Total genetic noise is controlled by induction. The total genetic noise, calculated as the standard error divided by the mean, is plotted for each of the conditions in Figure 2. Cyan corresponds to noise in cfp, yellow to noise in yfp, and red to noise in rfp. In each case, the noise is maximal in the fully induced state. The noise of each color is only affected by the associated inducer(s): aTc for cfp, IPTG for yfp, and both IPTG and L-ara for rfp. [file 1754-1611-4-10-S2.PDF]
